# Quantitative Chest X-ray Radiomics for Therapy Response Monitoring in Patients with Pulmonary Tuberculosis

**DOI:** 10.3390/diagnostics13172842

**Published:** 2023-09-01

**Authors:** Tamarisk Du Plessis, William Ian Duncombe Rae, Gopika Ramkilawon, Neil Alexander Martinson, Mike Michael Sathekge

**Affiliations:** 1 Department of Nuclear Medicine, Faculty of Health Sciences, University of Pretoria, Pretoria 0001, South Africa; 2 Medical Imaging Department, Prince of Wales Hospital, Sydney, NSW 2031, Australia; 3 Department of Statistics, Faculty of Natural and Agricultural Sciences, University of Pretoria, Pretoria 0081, South Africa; 4 Perinatal HIV Research Unit (PHRU), University of the Witwatersrand, Johannesburg 1862, South Africa; 5 Centre for Tuberculosis Research, Johns Hopkins University, Baltimore, MD 21205, USA

**Keywords:** radiomics, tuberculosis, segmentation, feature extraction, chest X-rays, radiomics score

## Abstract

Tuberculosis (TB) remains the second leading cause of death globally from a single infectious agent, and there is a critical need to develop improved imaging biomarkers and aid rapid assessments of responses to therapy. We aimed to utilize radiomics, a rapidly developing image analysis tool, to develop a scoring system for this purpose. A chest X-ray radiomics score (RadScore) was developed by implementing a unique segmentation method, followed by feature extraction and parameter map construction. Signature parameter maps that showed a high correlation to lung pathology were consolidated into four frequency bins to obtain the RadScore. A clinical score (TBscore) and a radiological score (RLscore) were also developed based on existing scoring algorithms. The correlation between the change in the three scores, calculated from serial X-rays taken while patients received TB therapy, was evaluated using Spearman’s correlation. Poor correlations were observed between the changes in the TBscore and the RLscore (0.09 (*p*-value = 0.36)) and the TBscore and the RadScore (0.02 (*p*-value *=* 0.86)). The changes in the RLscore and the RadScore had a much stronger correlation of 0.22, which is statistically significant (*p*-value *=* 0.02). This shows that the developed RadScore has the potential to be a quantitative monitoring tool for responses to therapy.

## 1. Introduction

Tuberculosis (TB) is a leading cause of death globally [1]. Planar chest X-rays (CXRs) are a method commonly used for early pulmonary TB (PTB) diagnosis in patients with clinical symptoms suggestive of TB. CXRs are used rather than more sophisticated three-dimensional modalities because they are the most widely accessible and inexpensive imaging modality in countries where TB is most prevalent [2,3,4]. Follow-up CXRs are also widely used in the management of PTB to evaluate clinical responses to treatment, though there are no objective tests for improvement. Intra- and interobserver discrepancies are common in X-ray reporting [5], and one study found an error rate of up to 23% when experienced radiologists reported on a collection of normal and abnormal X-rays [6].

For an assessment of responses to PTB treatment, in some settings, a follow-up CXR taken at the completion of the 2-month intensive four-drug treatment phase is often visually compared to the CXRs taken at the initiation of TB treatment [7]. With a subjective improvement in CXRs and symptoms, patients proceed to the two-drug continuation phase of TB treatment [7]. Currently, CXRs are only used for qualitative visual assessments in PTB management and are typically reported as either “improved” or “worse” compared to previous CXRs. However, if PTB characteristics can be quantified from chest X-rays, these characteristics might be used to evaluate disease response and could assist with identifying those who do not respond to TB treatment earlier in their treatment course.

Laboratory results are considered a ground truth for PTB management to determine responses to treatment [8]. However, in cases where laboratory results are not available, clinical signs and symptoms related to PTB are also a good indicator of treatment response. Scoring systems are tools designed to predict outcomes, assist in clinical decision-making, support treatment options and manage clinical risk [9]. Various clinical scoring algorithms for TB exist, and a systematic review study summarized the sensitivity and specificity of some of them [10]. The TBscore is one such system that was developed as a rapid and inexpensive tool to monitor TB patients in their treatment and to assess clinical outcomes [11]. This scoring system included five symptoms that were self-reported (cough, dyspnea, night sweats, haemoptysis and chest pain) and six signs that were assessed by a trained nurse (anemia, tachycardia, a positive finding on lung auscultation, fever, body mass index, and mid-upper arm circumference) [11]. This clinical score was also useful for TB diagnosis among adults living with HIV who presented to healthcare institutions with signs and symptoms suggestive of TB with high sensitivity (95.5%) and a specificity of 36.9% when a cut-off value of two was implemented [12]. In our study, we used this TB scoring system and modified it slightly for the population group under consideration.

Several radiological chest X-ray scoring systems have been reported, for example, the RALE (Radiographic Assessment of Lung Edema) score and the BRIXIA Chest X-ray Severity Scoring System [13]. Both scoring systems have lately been re-purposed for COVID-19 research [13,14,15,16]. In the RALE scoring system, each lung (left and right, respectively) is scored from 0 to 4, with 0 for no disease involvement and 4 for more than 75% involvement, with a total score out of 8 [13]. The BRIXIA score is used to grade lung abnormalities caused by disease on an 18-point severity scale [17]. This scoring system divides the lungs into 6 regions (3 regions for each lung), and each region is given a score (from 0 to 3) based on the extent of lung abnormalities detected [17]. For this retrospective study, we developed a TB radiological scoring system based on the aforementioned scoring systems that could be used to determine the correlation of newly developed scores.

Studies on the use of computer science from medical images for the diagnosis of PTB or on the differentiation of TB from another pulmonary disease have been reported [18,19,20], but very little evidence exists on the use of radiological image analysis for monitoring treatment response and prognosis [21]. As radiomics is gaining popularity in the medical field, it may be beneficial to use this to assess responses to TB therapy.

The motivation for our study was based on a recent study that developed a radiomics score (rad score) from CT scans which acted as a potential prognostic imaging feature for postoperative survival in solitary hepatocellular carcinoma (HCC) patients [22]. They identified six signature features and then applied the least absolute shrinkage and selection operator (LASSO) logistic model to develop the optimal rad-score algorithm [22]. This algorithm ensured that each feature contributed equally to the rad score since the dimensionality of features could differ significantly. This, in essence, is just a different approach to normalization. This study concludes that their rad score might be complementary to current tumor staging systems [22].

Most radiomic studies are performed using data from CT scans because of simplified disease segmentation and exposed (non-superimposed) anatomy. If PTB is the disease under investigation, CXRs would be more practical as they are an inexpensive, widely accessible imaging modality for disease diagnosis and management [4]. Our study, therefore, used a chest X-ray dataset of patients diagnosed with active PTB.

Some restrictions exist on performing radiomic feature extraction from planar chest X-rays, for example, the superimposed chest anatomy overlying the disease of interest and accurate disease segmentation to select the ROI for feature extraction [23]. To address this, a recent study developed a sliding window segmentation that was applied as a secondary segmentation to the entire segmented lung to include normal tissue and the disease [23]. This article shows that the sliding window segmentation could eliminate the need for accurate disease segmentation on planar images, as it produces accurate and reproducible radiomic signatures and models (AUC = 0.9444 (95% CI [0.8762, 0.9814])) [23]. This sliding window segmentation method was applied to our study, even though the traditional consequent steps of dimensionality reduction, signature development and model construction were not followed through.

In this study, a unique approach to dimensionality reduction and signature construction was applied to assess changes in CXRs that could be linked to other objective measures of TB treatment response using a novel CXR radiomics score. The correlation between the radiomics score and several calculated clinical- and radiological scores was evaluated.

## 2. Materials and Methods

### 2.1. Patient Selection

This was a secondary data analysis on the clinical- and radiological data of 111 patients collected for an observational study between August 2013 and July 2018. These patients had a laboratory-confirmed diagnosis of PTB with clinical data recorded and a CXR acquired at the baseline- and first follow-up clinic visit. The mean time lapse between the baseline and the first follow-up visit was 54.39 days (a range of 50–64 days), with all X-rays taken within one week of the date of the clinical visit. The CXRs were all reported by radiologists or attending doctors with extensive experience in PTB X-ray reporting.

### 2.2. Clinical Score (TBscore)

The TBscore algorithm was modified to calculate a clinical score for each patient [12]; it was minimally altered (see Table 1) to only include those signs and symptoms that were included in the initial clinical study database. Tolerances for BMI and pulse rate were intentionally altered by expert TB clinicians to have a similar quantitative contribution as the original TBscore. In the original TBscore algorithm, a BMI < 18 kg/m^2^ scored 1, a BMI < 16 kg/m^2^ scored 2, and a pulse rate of >90 bpm scored 1 with no value for score 2 [12]. 

In this study, four self-reported symptoms were included: general well-being (recorded as a self-reported answer to “how does the patient feel?” as either a score out of 10 (with 10 being “excellent” and 1 “awful”) or on a qualitative scale as “Good”, “Okay” or “Awful”), cough (the duration in days that coughing was experienced), BMI (body mass index in kg/m^2^) and night sweats (the number of days night sweats were experienced to date). Two clinical signs were included: temperature (oral or axillary temperature ≥ 37.5 °C) and pulse rate (in beats per minute) [12]. The symptoms and signs all had a possible score of 0, 1 or 2, resulting in a maximum clinical score out of 12 (see Table 1).

### 2.3. Radiological Score (RLscore)

The radiological dataset had the following 4 data entries recorded that were applicable to this study: (1) The CXRs’ acquisition date. (2) The presence of 4 radiological TB expressions (cavities, infiltrates, adenopathy and pleural effusion) in the left or right lung recorded separately. This was recorded as either ‘YES’ or ‘NO’, where ‘YES’ indicated disease presence and ‘NO’ indicated absent disease. For scoring purposes, binary values were assigned to the conditions in this study as YES = 1 and NO = 0. This added up to a maximum score out of 8 if all 4 expressions were present in both lungs. (3) The CXRs’ cavitation classification (recorded as 1, 2 or 3) were according to the conditions set out in Table 2. (4) Additionally, the extent of disease classification (recorded as A, B or C) was according to the conditions set out in Table 2. For scoring purposes, numerical values were assigned to each condition: A = 1, B = 2 and C = 3. Normal X-rays = 0.

A radiological score (RLscore) was obtained for each X-ray by including the recorded TB expressions (a possible 8 points), cavitation classification (a possible 3 points), and the extent of the disease (a possible 3 points). It was calculated according to the algorithm in Equation (1) for a maximum score out of 14.

(1) $$RLscore=Sum\left(Radiological\;TB\;expressions\right)+(Extend\;of\;disease)+(Cavitation\;classification)$$

### 2.4. Radiomics Score (RadScore)

**Figure 1:**
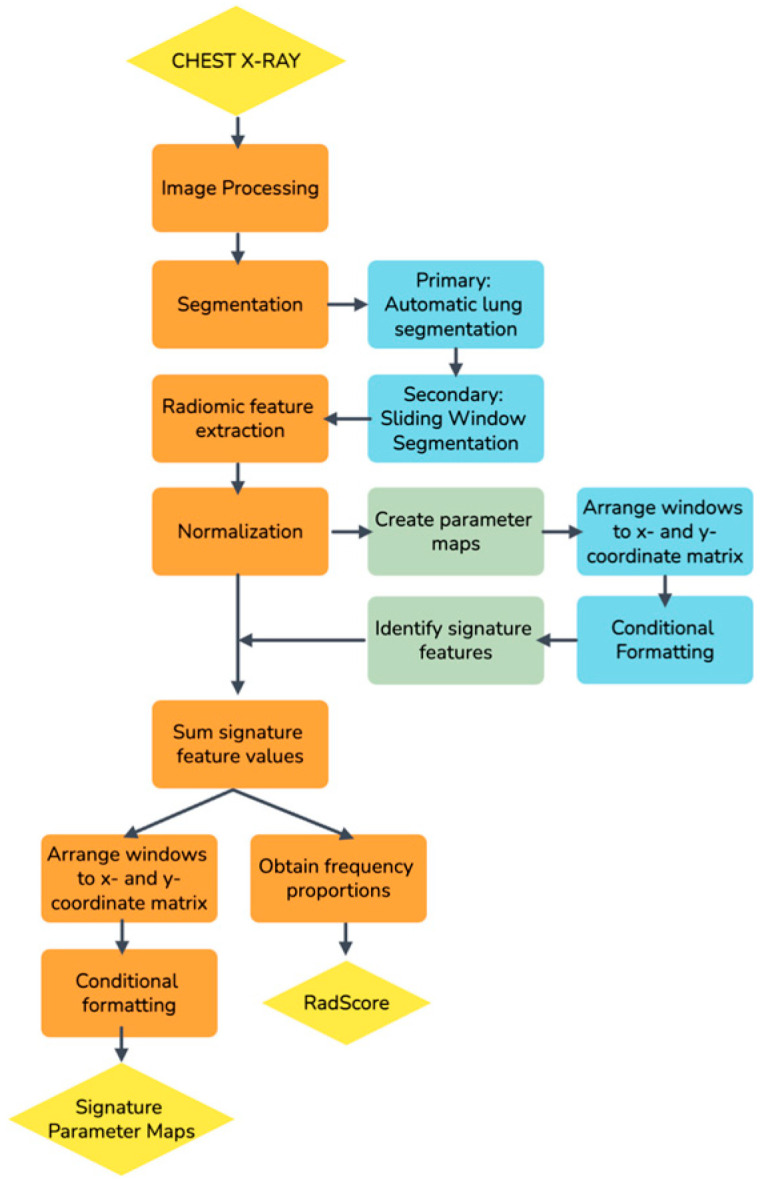
A schematic overview of the process followed to develop a radiomics score (RadScore) in this study.

A non-traditional method was followed to develop the radiomics score (RadScore) in this study when a sliding window segmentation was introduced [23]. Figure 1 is a schematic overview of the approach followed.

#### 2.4.1. Image Processing

The Total Image Converter (version 8.2.0.237) and Python (version 3.7.6) were used for initial pre-processing to ensure that a uniform CXR dataset was used as a cohort and that it was in the required format for the radiomics library. Image processing included manually cropping all images to square dimensions, correcting unconventional photometric interpretations on some images, converting DICOM images to the PNG format, interpolating all images to 256 × 256 pixels with bilinear interpolation, and converting the conventional RGB type of PNG images to the scalar type.

#### 2.4.2. Primary Segmentation

**Figure 2:**
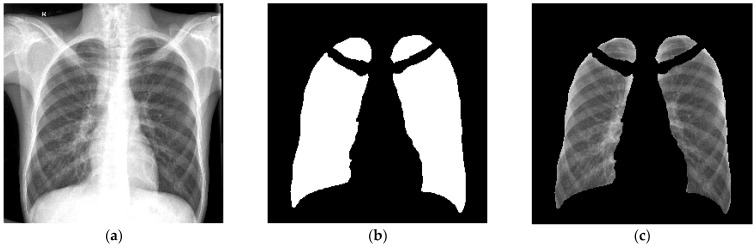
An example of the output of the primary segmentation model, (**a**) The original image, (**b**) The mask (multiplied by 255 to be visually visible) and (**c**) The mask multiplied with the original image. This image was used to evaluate the accuracy of the primary segmentation model [23].

A fully automatic in-house segmentation model was used to segment the lungs for the primary segmentation [24]. The segmentation model resized images to 256 × 256 pixels with bilinear interpolation before segmenting the lungs as a 256 × 256 pixel mask output [23]. Figure 2 is an example of the lung segmentation achieved with the clavicles removed.

#### 2.4.3. Secondary Segmentation and Radiomic Feature Extraction

Sliding window masks were created in Python (version 3.7.6) using Numpy.array() and PIL.Image() functions. A square sampling window mask (*w*) with 16 × 16 pixels and a window step size (*w_step_*) of 4 pixels was selected as has been previously described [23]. The window moved, or slid, from one side of the CXR to the other in both *x*- and *y*-dimensions to create a new window matrix of 61 × 61 windows. The number of windows in the matrix can be calculated using Equation (2) where [*P_x_*, *P_y_*] refers to the dimensions of the window matrix, [*n_x_*, *n_y_*] is the dimensions of the image in pixels, *w* is the window size and *w_step_* is the window step size [23].

(2) $$P_{x}=\frac{\left(n_{x}-w\right)}{w_{step}}+1\;AND\;P_{y}=\frac{\left(n_{y}-w\right)}{w_{step}}+1$$

The sliding windows, therefore, cause the effective dimensionality of each image’s features to increase by a factor of 3721 (61 × 61). This resolution was thought to be adequate to resolve the change in radiomic features across the lung within an acceptable computational time.

The sliding window masks were superimposed on the primary segmented lung mask of each CXR. Radiomic features were extracted from each window in the window matrix if the window was not masked off by the lung segmentation.

The Pyradiomics library (version 3.0) was used to extract 93 two-dimensional (2D) first-order and texture features from each sliding window on each CXR. Shape-based features were omitted, as these features used the ROI defined by the mask to calculate the values, which is meaningless for the purpose of this study.

#### 2.4.4. Creating Parameter Maps

**Figure 3:**
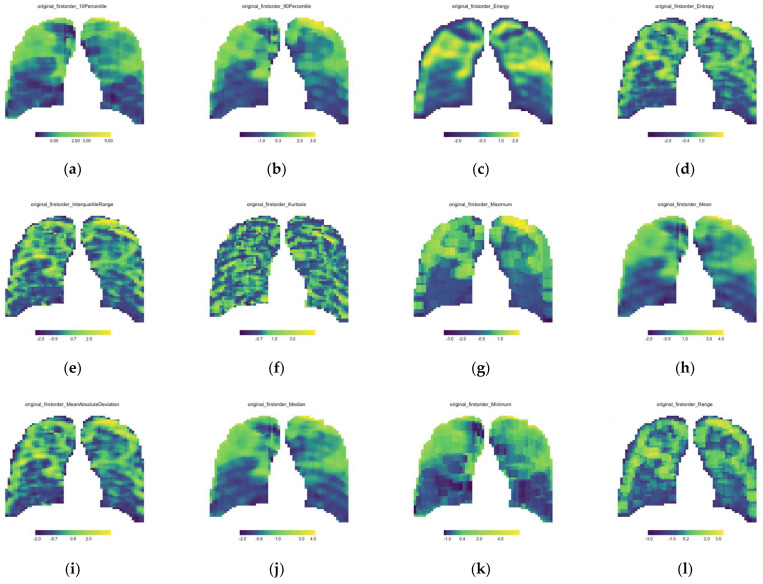

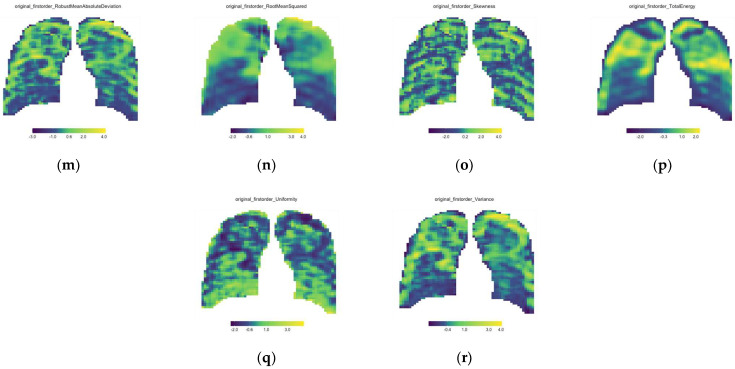
First order feature parameter maps for the single baseline CXR of Patient A; (**a**) 10th Percentile, (**b**) 90th Percentile, (**c**) Energy, (**d**) Entropy, (**e**) Interquartile range, (**f**) Kurtosis, (**g**) Maximum, (**h**) Mean, (**i**) Mean Absolute Deviation, (**j**) Median, (**k**) Minimum, (**l**) Range, (**m**) Robust Mean Absolute Deviation, (**n**) Root Mean Square, (**o**) Skewness, (**p**) Total Energy, (**q**) Uniformity and (**r**) Variance.

All data processing was performed using R Software (version 4.1.3). From Equation (2), it can be seen that 3271 window features were extracted per feature from each CXR. To account for the dimensional variability in these features, all windows’ features were normalized with z-score normalization. All feature values were redistributed to the correct *x*- and *y*-coordinates in the sliding window matrix. This was conducted for each of the 93 features extracted on each of the 111 CXRs. Heatmaps were applied, and a parameter map was printed for each feature (see Figure 3).

#### 2.4.5. Signature Features and Signature Parameter Map

A visual inspection of the parameter maps was the only method to identify the features that could result in a robust and reproducible radiomics signature to collate with the extent of the disease. Due to the enormous number of maps under investigation, it was decided to sample a simple random sample comprising more than 10% of the parameter maps. All parameter maps from this sample were visually inspected and compared to the original X-ray to identify the features that represented the disease. Six features were identified on 100% of the randomized samples’ CXRs, and these six features were acknowledged as the signature. The 6 signature feature values were summed to obtain a single signature value for each window in the parameter map out of a maximum of 6 (the sum of 6 normalized features). Since we noticed that these features all increased with an increase in disease status, we expected that the summed features would further highlight the diseased and the higher-density areas in the lung. When the 3721 values of the signature features were again redistributed to the correct *x*- and *y*-coordinates, a single signature parameter map of 61 × 61 windows for each CXR was developed.

#### 2.4.6. Developing the Radiomics Score (RadScore)

To develop a radiomics score that can be compared to a single integer radiology and clinical score, all the signature parameter maps were statistically summarized into a single value. The summed features for each CXR, with a numerical range from 0 to 6, were consolidated into four frequency bins with a bin width of 1.5. These 4 groups were [0.0; 1.5], (1.5; 3.0], (3.0; 4.5] and (4.5, 6.0]. The corresponding frequencies of the summed features for each group were calculated to obtain crucial information about where most feature information lies. Since the signature feature values increased with increased disease status, we could assume that a higher score indicated a worse state of PTB and would likely fall in the (3.0; 4.5] or (4.5, 6.0] frequency group with healthier lung tissues falling in the [0.0; 1.5] and (1.5; 3.0] groups. Due to dimensionality issues caused by the image segmentation techniques applied to each CXR, the summed features did not all contain information at the exact same windows from baseline to follow-up. The proportions were, therefore, used for each of the four groups to circumvent spurious results and to improve accuracy in comparisons between the baseline and follow-up CXRs.

### 2.5. Longitudinal Change

To evaluate the change in the RadScore, RLscore and the TBscore, the follow-up visit’s score was subtracted from the baseline visit’s score according to Equation (3).

(3) $$Change\left(Score\right)=First\;visit\left(Score\right)-Second\;visit(Score)$$

A positive change, therefore, indicates a radiological or clinical improvement, a negative change indicates worsening, and 0 indicates no change.

Spearman’s correlation analysis (the non-parametric equivalent of Pearson’s correlation analysis) was performed with a test of significance to evaluate the RadScore, TBscore and RLscore correlations. Spearman’s test was used since the clinical score was found to not be normally distributed at a 5% level with a *p*-value of 0.01 using the Shapiro–Wilk test of normality.

## 3. Results

### 3.1. Parameter Maps

Parameter maps are the normalized features extracted from each CXR’s sliding window mask and re-distributed into the correct spatial dimensions with conditional formatting applied. Figure 3 is only the 18 first-order features’ parameter maps of a single CXR (Patient A—Baseline CXR).

Visual evaluation was used to identify the features whose parameter maps correlated with disease pathology in the lungs. Table 3 provides a list of the six features identified as signature features. 

**Figure 4:**
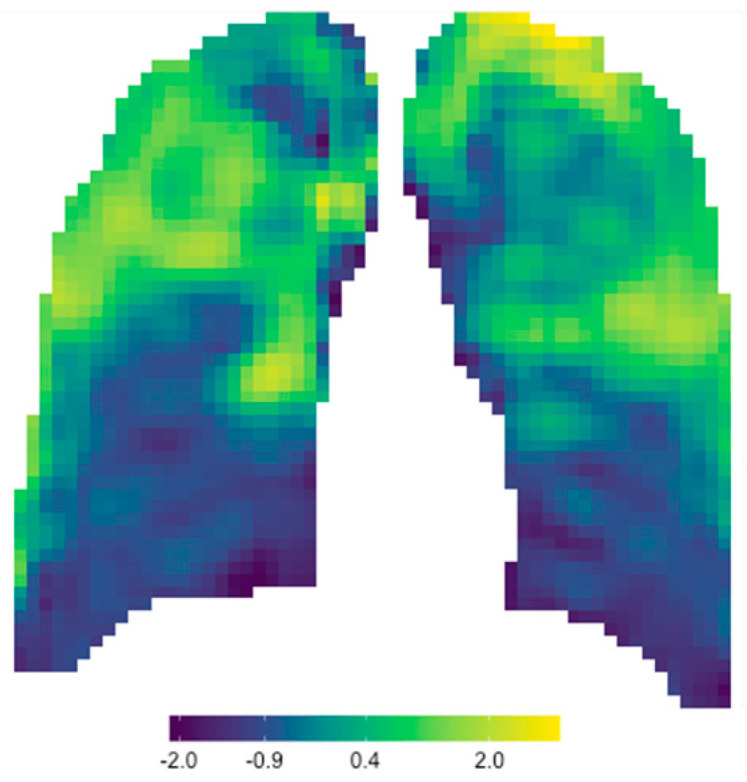
Signature parameter map obtained for Patient A’s baseline CXR.

To obtain signature parameter maps, the six signature features’ values were summed to obtain a signature value for every window out of six. These values were again re-distributed to the correct *x*- and *y*-coordinate matrix, and conditional formatting was applied to obtain a single signature parameter map for each CXR. Figure 4 is an example of the signature parameter map of Patient A’s baseline CXR (the same patient used in Figure 3).

### 3.2. Developing a RadScore

**Figure 5:**
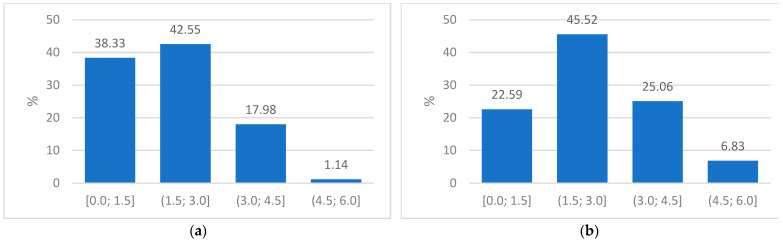
(**a**) Plot of frequency proportions in the baseline CXR of Patient A indicating the four groups used to obtain a radiomics score and (**b**) Plot of the frequency proportions in the corresponding follow-up CXR of Patient A.

Frequency bins were used to develop a RadScore for each patient at each clinical visit. Figure 5 shows the frequency bins of Patient A from their baseline and first follow-up CXR.

### 3.3. Change in RLscore, TBscore and RadScore

**Figure 6:**
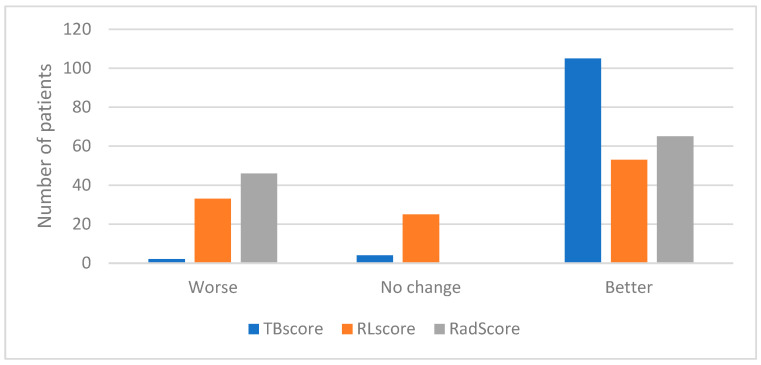
Graph indicating the number of patients who showed a decline, no change or an improvement in their TBscore, RLscore and RadScore.

Equation (3) was used to calculate the longitudinal change in the RLscore, TBscore and RadScore with the results shown in Figure 6.

**Figure 7:**
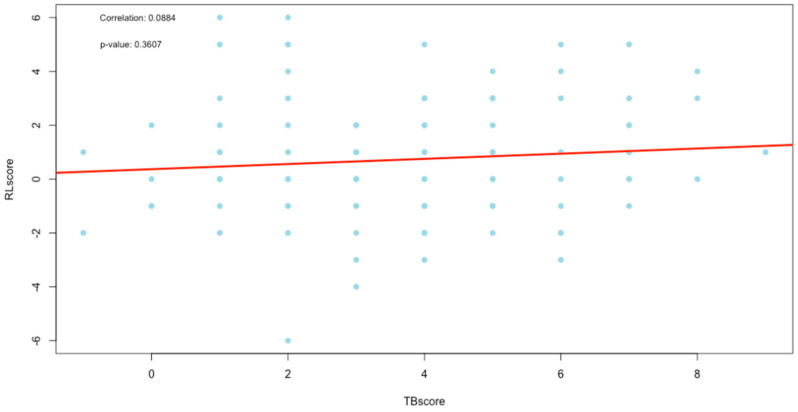
Plot of correlation between the TBscore and RLscore.

**Figure 8:**
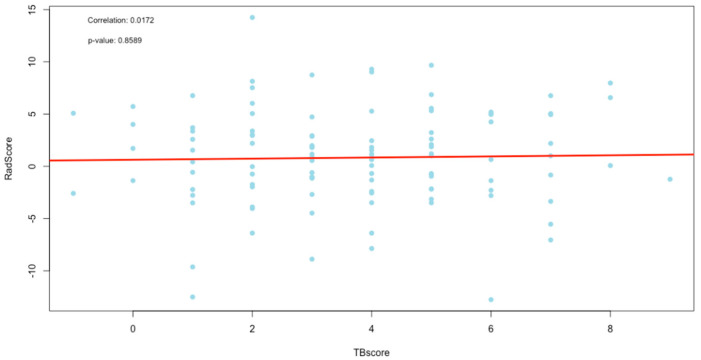
Plot of correlation between the TBscore and RadScore.

**Figure 9:**
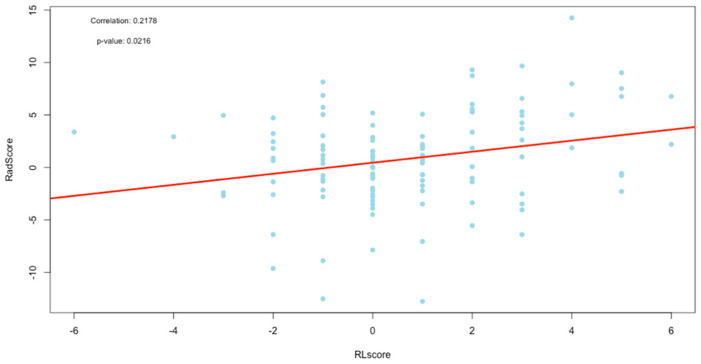
Plot of correlation between the RLscore and RadScore.

The correlation between the change in scores was evaluated using Spearman’s correlation with a test of significance for the correlation. The results are recorded in Table 4, Figure 7 (TBscore vs. RLscore), Figure 8 (TBscore vs. RadScore) and Figure 9 (RLscore vs. RadScore).

## 4. Discussion

In this study, we developed a TB radiomics score (RadScore) from chest X-rays without any need for disease segmentation. In traditional radiomic studies, the disease under investigation (mostly tumors) is precisely delineated, either manually or with an automatic or semi-automatic model [25,26]. The radiomic features are then extracted in the segmented ROI only, and a signature is developed that describes the properties of the delineated disease. However, the precise delineation of a non-neoplastic disease such as TB is very difficult. The sliding window method that was introduced as a secondary segmentation allowed us to evaluate the change in radiomic features across the entire lung region. By re-distributing every window’s extracted feature into the corresponding *x*- and *y*-coordinates, parameter maps could be developed. These parameter maps are, in essence, a quantified interpretation, either as a first- or second-order (texture features) statistical algorithm of the original image. We noticed that only first-order features’ parameter maps corresponded to lung pathology. This was expected as the texture features derived from secondary matrixes are too far removed from the original grayscale intensities of the CXR.

The parameter maps appeared as smoothed image versions because the sliding windows in the secondary segmentation reduced the resolution of the image. This segmentation allows for some overlap of the pixels from one window to the next, which ensures that no image information is lost. When the window size of the secondary segmentation is reduced, the signature parameter map seems less smooth, but this is at the cost of additional computational time and resources. The boundaries between the features in this study were well resolved as we used a window step size of only four pixels [23].

**Figure 10:**
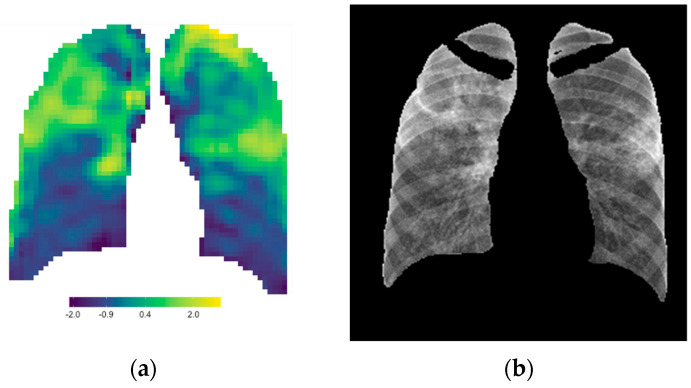
The radiomic signature parameter map (**a**) correlates strongly to the lung pathology on the CXR (**b**).

Signature parameter maps were developed in this study by adding the six features that individually corresponded to lung pathology. Other mathematical and statistical methods were also considered to develop a single signature value for each window, but it was found that the simple sum of these features was the optimal choice to highlight lung pathology compared to the standalone features’ parameter maps. Figure 10 shows the strong correlation between the radiomic signature map (a) and the original CXR (b). Since each window in the signature parameter map had a normalized quantitative signature value that was directly proportional to the color scale, this parameter map could be used not only as a visual tool enhancing radiological features but also as a quantitative tool to assist in CXR reporting, especially in disease management. When the parameter map of a baseline CXR is compared to that of a follow-up CXR, uncertainties in visual assessment can be eliminated using quantitative comparisons of the lung ROI.

If image registration was included as an initial step in this study, it might have been possible to use image subtraction of the baseline and follow-up CXR to determine differences in the signature maps. These differences would have clearly indicated the change in the extent of the disease (better or worse) and the position, visually and quantitatively. However, in this retrospective study, image registration was not considered. For individual cases, manual quantitative comparisons of the lung region of interest could be made, but this is too time-consuming to incorporate as a standard practice in the clinical environment.

The aim of our study was ultimately to develop a radiomics score that could automatically assign a single numerical value to each CXR and contribute to quantitative X-ray reporting, which is essential but still lacking for evaluating TB responses to treatment. Various consolidation methods were evaluated; however, we concluded that by using the change in the proportion of the windows (with feature values between 3.0 and 6.0), the most reliable score could be achieved. To evaluate the relevance of the developed radiomics score (RadScore), its correlation to a developed clinical- and radiological score was tested.

Figure 7, Figure 8 and Figure 9 are the plots of correlations, and visually, they seem to indicate the presence of outliers. The presence of outliers is known to be problematic, particularly within correlation analysis [27]. To this end, all outliers were tested to see if they were statistically significant using the Grubbs test [28]. Furthermore, the practical and clinical importance of these outliers were carefully considered as well. In all cases, the outliers were statistically insignificant and were, therefore, not removed.

The TBscore and the RLscore indicated a poor correlation of 0.09 with no statistical significance (*p*-value = 0.36). This might be accounted for by the slower improvement seen in lung pathology on a CXR compared to a faster response to treatment in the clinical signs and symptoms of PTB. Additionally, the lower sensitivity of X-rays in detecting minor changes in lung pathology can be a contributing factor [29]. This could also explain the even poorer correlation of 0.02 between the TBscore and the RadScore, with no statistical significance (*p*-value *=* 0.86). It is known that CXR plays a vital role in the management of PTB, but it cannot be used as a standalone tool [30]. The poor correlations between clinical signs and symptoms and the RL and RadScore also show that they cannot be used in isolation from laboratory-confirmed results in the management of PTB.

The RLscore and the RadScore had a much stronger correlation of 0.22, which was statistically significant (*p*-value *=* 0.02). This shows that the RadScore accurately quantifies the subjective radiological reports. The RadScore might, therefore, have the potential to eliminate the need for CXR reporting in TB management programs, which is currently only qualitative. This could benefit some of the world’s highest TB-burdened countries that have limited resources and a shortage of clinicians experienced in CXR reporting. If the RadScore is applied and supported with the developed signature parameter maps as a quantitative objective interpretation of CXR, it could improve the accuracy of X-ray reporting and strengthen the current role of CXRs in TB management programs.

Since the X-rays were used as a foundational input for the RadScore construction, an excellent correlation between the RL and RadScore was expected if dimensionality reduction and signature development were accurately applied, which we ensured in this study. The likely cause of the lower-than-expected correlation is the high-density ribs that were superimposed on the lung anatomy in the lung ROI. These higher-density overlying structures caused noise that was detected in the radiomic features. This reduced the accuracy of the features extracted, especially in the lateral lung regions, as seen in Figure 4. We attempted to address this issue by applying a bone suppression model, which was proven to outperform most other available models at the time [31], to all CXRs in this study before the primary lung segmentation. This was, however, unsuccessful as this model was still too immature at the time of this study due to the small training cohort, with a limited dynamic range, that was used to train this model. When a matured and accurate bone suppression model is developed that can retrospectively remove the ribs, most of the noise that influences the radiomic features will be removed. The radiomics scoring method proposed in this study would then most likely result in a RadScore that has a very high correlation to the RLscore.

The methodology introduced in this study is labor-intensive and expensive in terms of knowledge and computational resources. A future perspective is to develop this into software with a user-friendly interphase where a CXR image can easily be uploaded and the clinical data entered. The software can then automatically perform the image post-acquisition processing, apply the segmentation model and radiomic feature extraction library to the CXR, and complete the rapid parameter map and score constructions. If this is developed as an application rather than server-based software, it should be accessible from any computer, regardless of its processing power. This eliminates the restriction of computational power, which might be a limiting factor in many high-TB-burdened countries. Following this, RadScore should be useful as a quantitative tool to evaluate the changes in PTB disease characteristics as seen from CXRs, which could greatly assist clinicians, especially in resource-limited countries, in the management of PTB.

## 5. Conclusions

In this study, a radiomics score (RadScore) was developed from chest X-rays that showed a good correlation, with statistical significance, to a developed radiological score. It was shown that the RadScore can be used to quantify the change in the disease characteristics seen on X-rays of patients diagnosed with pulmonary TB. Integrating the RadScore as a quantitative objective interpretation of X-rays could improve the accuracy of X-ray reporting and strengthen its role in TB management programs. As part of the RadScore construction, signature parameter maps were created that showed excellent qualitative correlations that could further increase the acceptance of chest X-rays as a quantitative tool for assessing TB response in medical research and clinical practice.

## Figures and Tables

**Table 1:** Clinical score chart used in this study with a maximum score of 12.

Sign or Symptom	Criteria		
Score	0	1	2
How do you feel?	7–10 (Good)	4–6 (Okay)	≤3 (Awful)
Fever (°C)	≤37.5 (NO)		>37.5 deg (YES)
Pulse rate (bmp)	<90	90–100	>100
Cough (days)	No cough	<60 or No cough at prior visit	≥60 or No cough at prior visit
BMI (kg/m^2^)	>20	18–20	<18
Nights sweats (days)	0	<60 days	≥60 days

°C = Degrees Celsius, bpm = beats per minute, kg/m^2^ = kilogram per meter squared.

**Table 2:** Radiological score conditions of the X-ray classification.

X-ray Classification: Cavitation		X-ray Classification: Extent of Disease	
1	Absent, as seen on a posteroanterior (PA) or anteroposterior (AP) CXR view.	A (1)	Limited: Lesion(s) involving a total lung area less than one-quarter of the area for the entire thoracic cavity, as seen in the PA or AP view.
2	Single or multiple cavities with diameter < 4 cm in aggregate (for each cavity, measure at point of maximum diameter) for a PA or AP CXR view.	B (2)	Moderate: Lesion(s) greater than A, but which, even if bilateral, involve a total lung area of less than one-half the area of the entire thoracic cavity, as seen in the PA or AP view.
3	Single or multiple cavities with diameter ≥ 4 cm in aggregate (each cavity was measured at the point of maximum diameter) for a PA or AP CXR view.	C (3)	Extensive: Lesion(s) involving a total lung area equal to, or more than half the area, of the entire thoracic cavity, as seen in the PA or AP view.

**Table 3:** The following features were identified as signature features that highlight and quantify the disease and correlate to the lung pathology.

Feature Number	Feature Name
1	First order—90th percentiles
2	First order—Median
3	First order—Mean
4	First order—Energy
5	First order—Root mean square
6	First order—Total Energy

**Table 4:** The correlation between the change in the TBscore, the RLscore and RadScore as calculated using Spearman’s correlation analysis with *p*-value testing the significance of the correlation.

Scores Being Compared	Correlation Value	*p*-Value
TBscore vs. RLscore	0.0884	0.3607
TBscore vs. RadScore (prop. 3 to 6)	0.0172	0.8589
RLscore vs. RadScore (prop. 3 to 6)	0.2178	0.0216

## Data Availability

Data are available on request from the corresponding author.
